# Efficacy of encapsulated fecal microbiota transplantation and FMT via rectal enema for irritable bowel syndrome: a double-blind, randomized, placebo-controlled trial (CAP-ENEMA FMT Trial)

**DOI:** 10.3389/fmed.2025.1648944

**Published:** 2025-09-23

**Authors:** Natsuda Aumpan, Soonthorn Chonprasertsuk, Bubpha Pornthisarn, Sith Siramolpiwat, Patommatat Bhanthumkomol, Navapan Issariyakulkarn, Pornpen Gamnarai, Phubordee Bongkotvirawan, Arti Wongcha-um, Varocha Mahachai, Ratha-korn Vilaichone

**Affiliations:** ^1^Center of Excellence in Digestive diseases and Gastroenterology Unit, Department of Medicine, Thammasat University, Pathumthani, Thailand; ^2^Department of Medicine, Chulabhorn International College of Medicine (CICM) at Thammasat University, Pathumthani, Thailand; ^3^Department of Biochemistry, Faculty of Medicine, Thammasat University, Pathumthani, Thailand

**Keywords:** fecal microbiota transplantation, irritable bowel syndrome, capsule, rectal enema, fecal transplant

## Abstract

**Introduction:**

Irritable bowel syndrome (IBS) is a functional bowel disorder. Gut dysbiosis involves in pathogenesis of IBS. Limited studies compared efficacy of fecal microbiota transplantation (FMT) via different routes of administration. This study aimed to compare efficacy of encapsulated FMT, FMT via rectal enema, and placebo in IBS patients.

**Methods:**

In this double-blind, randomized, placebo-controlled study, we enrolled patients aged 18–70 years with IBS defined by Rome IV criteria at Thammasat university, Thailand. Patients were randomized into three groups: (1) encapsulated FMT (six capsules twice daily for two consecutive days, total 50 g of stool), (2) FMT via rectal enema (50 g of stool in 200 mL of isotonic saline), or (3) placebo. Primary endpoint was clinical response defined by ≥50-point decrease in IBS-symptom severity score (IBS-SSS) at 4 weeks. Secondary outcomes were quality of life and changes of fecal microbiota composition after treatment. The study was registered with ClinicalTrials.gov, number NCT06201182.

**Results:**

From August 20, 2020, to February 15, 2024, 45 patients were randomized to receive encapsulated FMT (*n* = 15), FMT via rectal enema (*n* = 15), or placebo (*n* = 15). There was no difference in patient characteristics and baseline IBS-SSS between groups. Encapsulated FMT provided significantly improved IBS-SSS (166.7 ± 73.7 vs. 269.3 ± 69.5, *p* = 0.001), clinical response (86.7 vs. 26.7%, *p* = 0.001), and quality of life (31.7 ± 4.8 vs. 25.1 ± 5.2, *p* < 0.001) at 4 weeks compared with placebo. FMT via rectal enema demonstrated better IBS-SSS (168.7 ± 101.9 vs. 269.3 ± 69.5, *p* = 0.004), clinical response (73.3 vs. 26.7%, *p* = 0.011), and quality of life (30.2 ± 5.0 vs. 21.0 ± 7.4, *p* < 0.001) than placebo. Clinical response and quality of life between encapsulated FMT and FMT via rectal enema were not different. No serious adverse event was observed. Minor adverse events such as bloating and diarrhea were not different between all groups.

**Conclusions:**

Higher clinical response and quality of life were demonstrated in both FMT groups than placebo. Either encapsulated FMT or FMT via rectal enema was safe and could provide favorable outcomes for IBS patients.

**Clinical trial registration:**

https://clinicaltrials.gov/study/NCT06201182, Identifier: NCT06201182.

## 1 Introduction

Irritable bowel syndrome (IBS) is a functional bowel disorder defined by recurrent abdominal pain related to defecation or a change in bowel habit (1). IBS is a common disorder with the global prevalence of ~10% (2). Patients can be categorized into three main subtypes according to their bowel habit predominance: IBS with constipation (IBS-C), IBS with diarrhea (IBS-D), and IBS with mixed bowel habits (IBS-M) (1). The pathogenesis of IBS include gut microbial dysbiosis, low-grade mucosal inflammation, increased gut permeability, and altered gut-brain interaction (3). Dysbiosis, which is gut microbiota imbalance, can contribute to IBS by compromising epithelial barrier integrity, modulating enteroendocrine signaling, and overstimulating mucosal immune system (4). Homeostasis of neural, endocrine and immune communication pathways in the gut-brain axis is predominantly modulated through activated vagus nerve by gut microbes and metabolites. Moreover, proteases derived from altered gut microbiota might lead to intestinal barrier dysfunction (5). Correcting dysbiosis by modulation of intestinal microbiota is an emerging treatment approach for IBS (6). Dietary adjustment and probiotics can alter gut microbiota and may be tried despite yielding inconsistent results (4, 7). Fecal transplantation, which previously resulted in successful treatment of *Clostridioides difficile* infection (8), has been more popularly used for treatment of other diseases linked to gut dysbiosis.

Fecal microbiota transplantation (FMT) is an administration of feces from a healthy donor to the gastrointestinal tract of a recipient to restore balanced microbial flora (9). FMT could provide clinical response in IBS patients as demonstrated in some previous randomized controlled trials (10–12), whereas others reported no different outcome from placebo (13, 14). Most trials delivered FMT via more invasive routes such as colonoscopy or gastroscopy (10–12). There were few studies using less invasive methods, e.g., oral FMT capsules and FMT via rectal enema. Although two studies applying oral FMT capsules in IBS patients could not provide symptom relief, one study showed long-term gut microbiota alteration after FMT (13, 14). Appropriate treatment dose and delivery routes of FMT are required to be determined to achieve the best outcome in IBS patients. Moreover, less invasive routes of FMT administration are needed to be evaluated to reduce risk and cost of endoscopy.

Relieving symptoms and improving quality of life are essential for treatment of IBS (7). However, limited pharmacological treatment and inability to maintain constant dietary restriction might result in unsuccessful treatment outcome. Correcting gut dysbiosis might be another target to improve symptoms. Until now, there have been only a few studies using FMT via less invasive routes in patient with IBS. This study aimed to determine efficacy of FMT via rectal enema and encapsulated FMT in IBS patients in Thailand.

## 2 Materials and methods

### 2.1 Study design and participants

This was a double-blind, randomized, placebo-controlled study conducted at Thammasat university, Thailand between August 20, 2020 and February 15, 2024. The inclusion criteria were patients aged between 18 and 70 years with IBS defined according to Rome IV criteria (1). The exclusion criteria were pregnancy, breastfeeding, fecal incontinence, severe comorbidities (end-stage renal disease, Child-Pugh class C cirrhosis, acquired immunodeficiency syndrome, cancer, cerebrovascular disease, cardiovascular disease), immunodeficiency disorders, use of immunosuppressive drugs, use of probiotics within 4 weeks prior to study entry, or use of IBS medication within 4 weeks prior to study entry. Written informed consent was obtained from all patients and donor prior to enrollment. This study was conducted according to the good clinical practice guideline, as well as the Declaration of Helsinki. The study was approved by the Ethics Committee of Thammasat University (MTU-EC-IM-1-080/63) and was registered with ClinicalTrials.gov (NCT06201182).

### 2.2 Randomization and masking

The researcher who was not involved in the clinical performance of the trial generated the randomization sequence with a block size of six using a web-based randomization. Random allocation sequences were in sealed opaque envelopes. Each patient had the study number according to the first available slot for treatment procedure. All patients and investigators were blinded to treatment allocation. Patients were randomized into three groups: (1) encapsulated FMT, (2) FMT via rectal enema, or (3) placebo at a ratio of 1:1:1. After FMT, patients were followed up at 2, 4, 8, and 12 weeks. The randomization key was revealed to investigators and patients after completion of 3-month follow-up.

### 2.3 Fecal microbiota transplantation procedure

#### 2.3.1 Donor screening

Participants aged 18–50 years without comorbidity were screened according to the guideline for stool donor (9). Fecal donors were subjected to physical examination and screened for various communicable diseases before being recruited to this study. The exclusion criteria were as follows: high risk of infectious diseases, inflammatory bowel disease, IBS, chronic constipation, chronic diarrhea, celiac disease, atopic conditions, autoimmune conditions, chronic pain syndromes, obesity, metabolic syndrome, psychiatric conditions, neurological disorders, malignancy, history of abdominal surgery, use of antibiotics in 3 months prior to stool donation. Blood tests were negative for HIV, *Treponema pallidum*, and hepatitis A, B, and C. Stool samples were also negative for *Helicobacter pylori, Clostridioides difficile, Salmonella* species (spp.), *Shigella* spp., *Campylobacter* spp., *Vibrio* spp., Cryptosporidium, viruses (Norovirus, severe acute respiratory syndrome coronavirus 2, Adenovirus, Rotavirus), and parasites. For detection of *C. difficile*, glutamate dehydrogenase (GDH), *C. difficile* toxin A and B immunoassay, and polymerase chain reaction (PCR) were performed. All laboratory tests used for donor screening were described in Supplementary Table 1. This study used stool samples from a single donor, a 24-year-old healthy female without underlying medical condition. She was born via a vaginal delivery and breastfed. Her body mass index was normal as 20.3 kg/m^2^. After recruitment, the donor maintained a healthy lifestyle by consumption of high-fiber low-fat diet and regular moderate-intensity exercise 1–1.5 h per day for 5 times per week. Fecal microbiota profile demonstrated that dominant phyla based on relative abundance were Firmicutes (57%), Bacteroidota (24%), and Actinobacteriota (18%). During stool donation, the donor was asked to stay on stable healthy diet and scheduled for a regular blood and stool screening program every 6 months.

#### 2.3.2 Preparation of frozen stool and placebo

Fecal samples from a healthy donor were stored in −80 °C freezer. Samples for rectal enema and capsules were prepared on the day of FMT procedure by an independent staff who was not involved in the clinical performance of the trial. The dose of 50 grams of stool (15) diluted to 250–500 ml of infusate is commonly used in most studies (16). When using for rectal enema, 50 grams of frozen stool samples were thawed in a warm water bath (37 °C) for 1–2 h. Stool was subsequently mixed with 200 ml of isotonic saline and stool suspension was filtered through a double layer of standard micropore to remove debris. Stool suspension was filled in four 50-ml syringes covered by opaque paper and syringe tip caps. Placebo enema was also prepared using 200 ml of isotonic saline in four syringes covered by opaque paper and syringe tip caps. Isotonic saline was chosen as an inert placebo in our study which was similar to the previous study (17). FMT or placebo was applied via rectal enema within 2 h after preparation. For encapsulation, 50 grams of frozen stool samples were introduced in the lyophilizator (Lyo-Works^TM^ Operating System Freeze Dryer, Kansas city, MO, USA) with a shelf temperature of −50 °C under vacuum pump with a displacement of at least 86 liters per minute and 0.002 mBar ultimate pressure for 48 h. After lyophilization, freeze-dried fecal powder was filled in 24 capsules and stored in −80 °C freezer until the day of delivery. FMT and placebo capsules were identical in appearance including color, consistency, and odor.

#### 2.3.3 Interventions

Patients were scheduled for rectal enema administration at the day procedure unit. The procedure was performed by a researcher who was not involved in the clinical performance of the trial. A patient was asked to lie on his left side with knees flexed toward his chest, and then a rectal tube was inserted. FMT or placebo solution was administered by four completely covered syringes via rectal tube. The foot end of the bed was slightly elevated to approximately 20–30 degrees. Patients were requested to stay on their left side for 10 min, then change to supine position for 10 min, and lie on right side for another 10 min to increase colonic distribution. After finishing rectal enema procedure, patients were given 24 FMT or placebo capsules and instructed to take six capsules twice daily for two consecutive days. The first six capsules were taken within 2 h after rectal enema procedure. All patients were instructed to keep capsules in the freezer. Patients in FMT enema group received 200 ml of FMT solution via rectal enema and placebo capsules, whereas patients in encapsulated FMT group received FMT capsules and 200 ml of isotonic saline. Patients in the placebo group received 200 ml of isotonic saline and placebo capsules.

### 2.4 Microbiome analysis

DNA was extracted from fecal samples employing QIAamp PowerFecal Pro DNA Kits. Full-length 16S rRNA was amplified via PCR using the forward primer S-D-Bact-0008-c-S-20 (5′-AGRGTTYGATYMTGGCTCAG-3′) and the reverse primer 1492R (5′-CGGYTACCTTGTTACGACTT-3′) (18). DNA libraries were then prepared and sequenced utilizing MinION technology. Full-length 16S rDNA data were obtained in the FAST5 format, subsequently converted into FASTQ format using Guppy v7.1.4. Sequencing data underwent filtration based on length and base quality criteria using Filtlong v0.2.1, with parameters set as min_length = 1,300, max_length = 1,700, and min_mean_q = 20. The Emu pipeline was employed for the classification and quantification of sequences into taxonomies, leveraging the SILVA database v138 (19). Rarefying normalization was conducted at a read depth of 100,000 using the vegan v2.6-4 library. Microbiome indices, encompassing alpha diversity and beta diversity, were computed utilizing the vegan v2.6-4 and phyloseq v1.46.0 libraries.

### 2.5 Outcomes

The primary outcome was clinical response defined by a decrease in IBS-symptom severity score (IBS-SSS) by ≥50 points at 4 weeks after FMT. The IBS-SSS is a validated composite score containing five questions rated on a 100-point visual analog scale (total score of 0–500) (20). Five questions include abdominal pain, number of days with abdominal pain in the past 10 days, abdominal distension, satisfaction with bowel habits, and interference of daily life in the past 10 days. IBS-SSS was recorded at baseline before FMT, 2, 4, 8, and 12 weeks after FMT.

Secondary outcomes were (1) changes in quality of life (QOL) score after treatment; (2) adverse events during and after treatment; and (3) changes of fecal microbiota composition before and after treatment. QOL scores were assessed by a 16-item measure composed of physical domain, psychological domain, social relationship, and environment related to patients' IBS symptoms. Each item is rated from 0 to 3. QOL scores were recorded at baseline before FMT, 4, and 12 weeks after FMT. Patients' stool before and 4 weeks after FMT was collected for microbiome analysis.

### 2.6 Statistical analysis

The sample size required in each arm of the trial was calculated by assuming that placebo and FMT effect response at 4 weeks was 25.5 and 75.9%, respectively according to the previous study (10). The total sample size was estimated to be 45 patients with 15 in each arm with a power (1 – β) of 0.80 and an alpha of 0.05 (two-tailed test). All data were analyzed by using SPSS version 22 (SPSS Inc., Chicago, IL, USA) and R version 4.3.1 (RStudio Inc., Boston, MA, USA). Categorical variables were analyzed by Chi-square test, or Fisher's exact test where appropriate. The differences between placebo, FMT via rectal enema, and encapsulated FMT in gender, comorbidities, IBS subtypes were analyzed by Chi-square test. Continuous variables such as age, IBS-SSS, and QOL score were analyzed by using Student's *t*-test and reported as mean ± standard deviation (SD). Kruskal-Wallis one-way analysis of variance (ANOVA) was performed to compare continuous data among three groups. Quality of life scores before and after FMT were compared using paired *t*-test. For microbiome analysis, statistical analyses among either conditions or treatment method were carried out using the paired Wilcoxon rank-sum test and Kruskal-Wallis test. All visualizations were generated using the ggplot2 v3.4.4 library. All tests were two-sided and *p*-values of <0.05 were considered as statistical significance.

## 3 Results

### 3.1 Baseline characteristics

**Figure 1:**
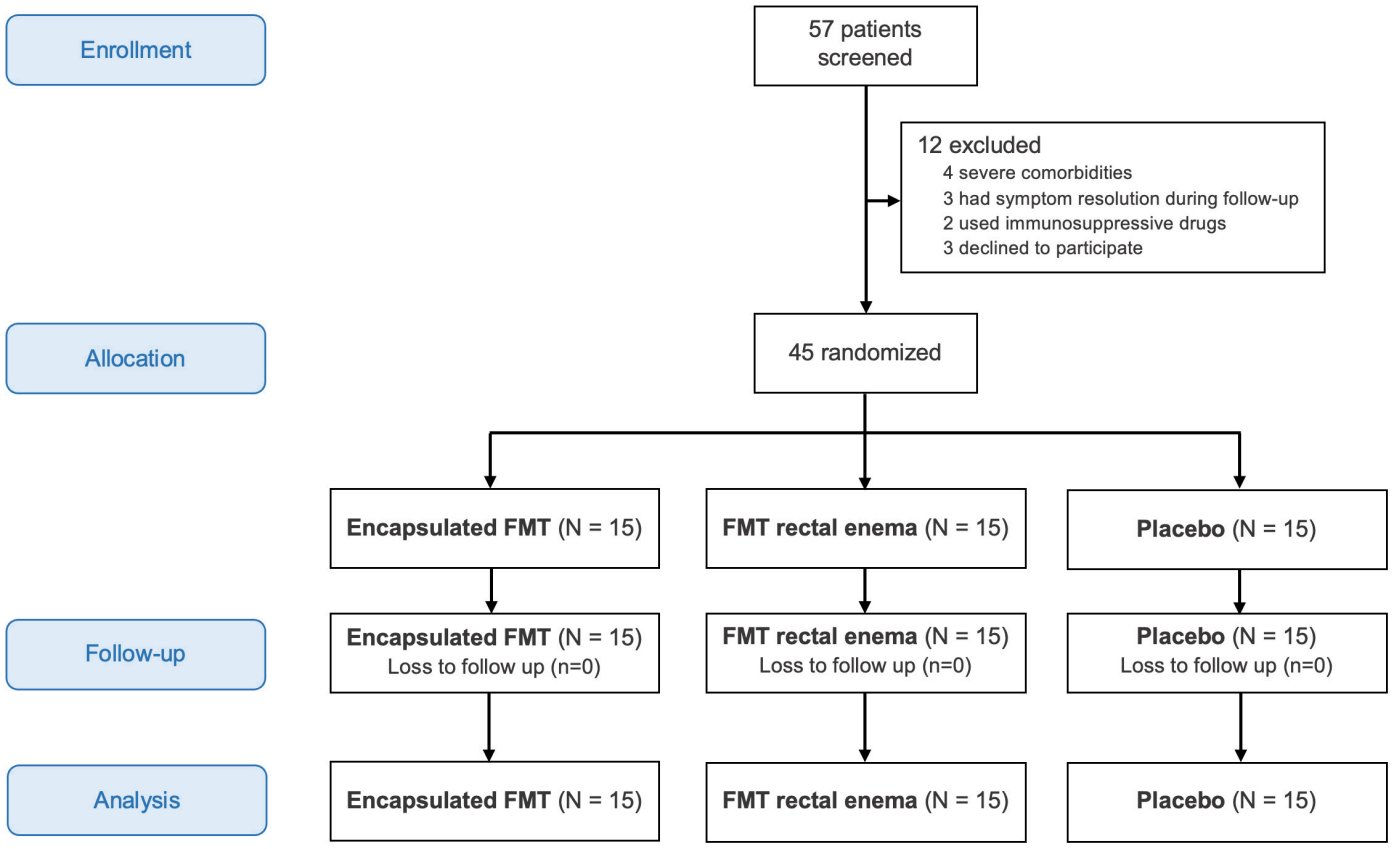
CONSORT flow diagram.

Between August 20, 2020 and February 15, 2024, 57 patients with IBS were assessed for eligibility. Twelve were excluded from this study because of severe comorbidities, symptom resolution during follow-up, immunosuppressive drug use, or refusal to participate in the study (Figure 1). Forty-five patients were randomized into three groups to receive encapsulated FMT, FMT via rectal enema, or placebo. There were 15 patients in each group without dropout. The mean age of all patients was 49.6 years and 37.8% were males. Patient characteristics were demonstrated in Table 1. Most patients did not have any comorbidity (42.2%). The most common comorbidities were dyslipidemia (26.7%) followed by hypertension (11.1%). IBS subtypes of all patients in this study were IBS-C (51.1%), IBS-D (24.4%), and IBS-M (24.4%). Baseline demographic data and IBS subtypes were not different among three groups.

**Table 1:** Patient characteristics.

**Characteristics**	**Total (*N* = 45)**	**FMT capsules (*N* = 15)**	**FMT rectal enema (*N* = 15)**	**Placebo (*N* = 15)**	***P*-value**
Gender, male	17 (37.8%)	7 (46.7%)	4 (26.7%)	6 (40%)	0.516
Age, years (mean ± SD)	49.6 ± 14.6	51.0 ± 16.3	48.1 ± 14.1	49.7 ± 14.2	0.804
BMI, kg/m^2^ (mean ± SD)	23.0 ± 3.3	22.6 ± 2.9	23.1 ± 2.5	23.4 ± 4.6	0.863
**Comorbidities**					
None	19 (42.2%)	6 (40.0%)	5 (33.3%)	8 (53.3%)	0.529
Dyslipidemia	12 (26.7%)	5 (33.3%)	4 (26.7%)	3 (20.0%)	0.711
Hypertension	5 (11.1%)	1 (9.1%)	3 (20.0%)	1 (9.1%)	0.407
Diabetes mellitus	4 (8.9%)	1 (6.7%)	2 (13.3%)	1 (6.7%)	0.760
Hepatitis	4 (8.9%)	1 (6.7%)	1 (6.7%)	2 (13.3%)	0.760
Psychiatric comorbidity	9 (20%)	4 (26.7%)	3 (20.0%)	2 (13.3%)	0.659
IBS subtype					0.772
IBS-C	23 (51.1%)	7 (46.7%)	7 (46.7%)	9 (60.0%)	
IBS-D	11 (24.4%)	3 (20.0%)	4 (26.7%)	4 (26.7%)	
IBS-M	11 (24.4%)	5 (33.3%)	4 (26.7%)	2 (13.3%)	
IBS-SSS at inclusion	322.9 ± 48.4	316.0 ± 37.8	329.3 ± 63.1	318.7 ± 40.2	0.752

FMT, Fecal microbiota transplantation; BMI, Body mass index; IBS, Irritable bowel syndrome; IBS-C, IBS with predominant constipation; IBS-D, IBS with predominant diarrhea; IBS-M, IBS with mixed bowel habits; IBS-SSS, IBS symptom severity score.

### 3.2 Symptom severity

**Figure 2:**
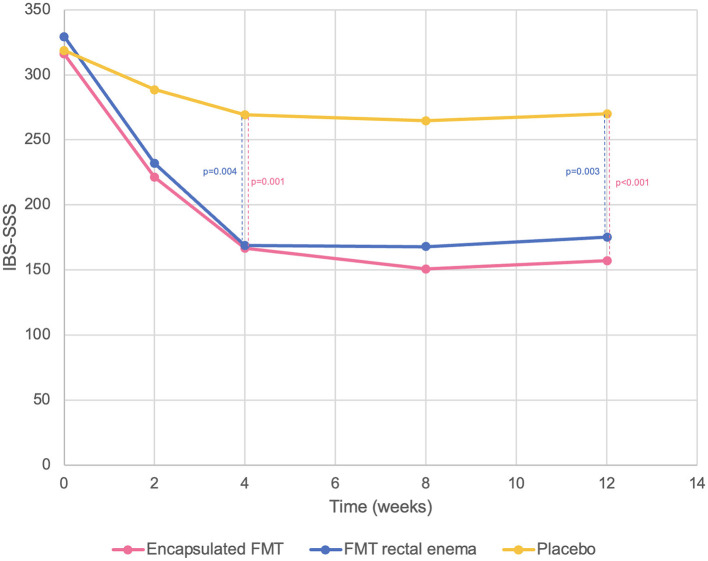
IBS-SSS compared between encapsulated FMT, FMT rectal enema, and placebo.

**Figure 3:**
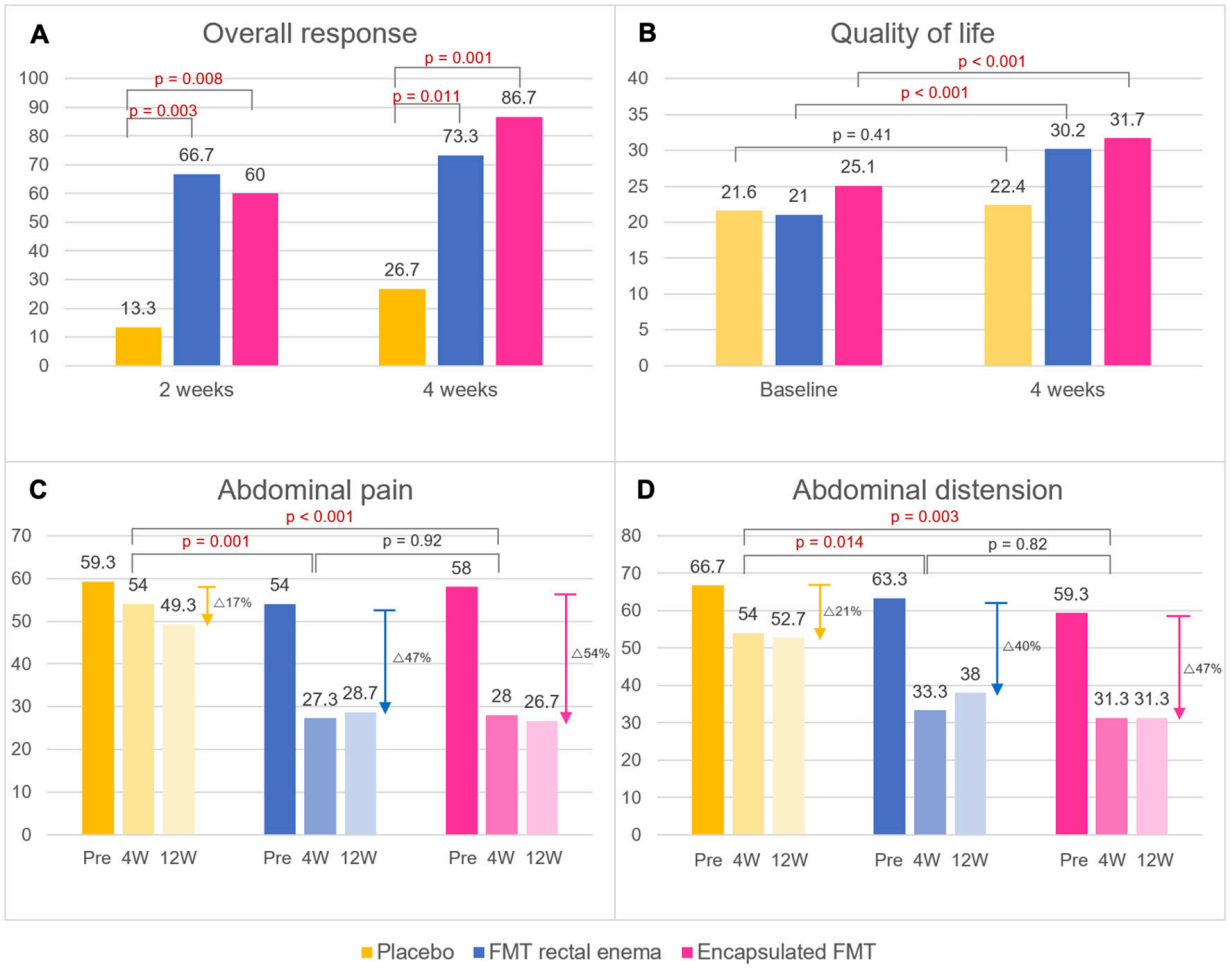
Primary and secondary endpoints after FMT. **(A)** Overall response, **(B)** quality of life scores, **(C)** abdominal pain scores, **(D)** abdominal distension scores. Data are expressed as mean. W, weeks.

There was no difference in baseline IBS-SSS at inclusion in all groups (encapsulated FMT 316.0 ± 37.8 vs. FMT rectal enema 329.3 ± 63.1 vs. placebo 318.7 ± 40.2, *p* = 0.752). Encapsulated FMT provided a significant improvement of IBS-SSS (166.7 ± 73.7 vs. 269.3 ± 69.5, *p* = 0.001) and overall clinical response (86.7% vs. 26.7%, *p* = 0.001) at 4 weeks compared with placebo. FMT via rectal enema also demonstrated better IBS-SSS (168.7 ± 101.9 vs. 269.3 ± 69.5, *p* = 0.004) and clinical response (73.3 vs. 26.7%, *p* = 0.011) at 4 weeks than placebo. At 12 weeks after FMT, patients who received FMT capsules still had significantly higher IBS-SSS (157.3 ± 75.3 vs. 270.0 ± 63.0, *p* < 0.001) and overall clinical response (86.7 vs. 33.3%, *p* = 0.003) than placebo group. IBS-SSS (175.3 ± 91.3 vs. 270.0 ± 63.0, *p* = 0.003) and clinical response (73.3 vs. 33.3%, *p* = 0.028) in the FMT rectal enema group were also better than placebo at 12 weeks. However, IBS-SSS and clinical response between encapsulated FMT and FMT via rectal enema were not different at 4 and 12 weeks after FMT. Classified by IBS subtypes, patients with IBS-D receiving FMT had significantly higher clinical response at 4 weeks than placebo (100 vs. 0%, *p* = 0.003), whereas patients with IBS-C and IBS-M receiving FMT had numerically higher clinical response (IBS-C 64.3 vs. 33.3%, *p* = 0.214; IBS-M 88.9 vs. 50%, *p* = 0.345). For different symptom dimensions of IBS, abdominal pain (28.0 ± 15.7 vs. 54.0 ± 17.2, *p* < 0.001) and abdominal distension scores (31.3 ± 22.0 vs. 54.0 ± 16.4, *p* = 0.003) significantly decreased at 4 weeks in encapsulated FMT group than placebo. FMT via rectal enema also provided significantly improved abdominal pain (27.3 ± 20.2 vs. 54.0 ± 17.2, *p* = 0.001) and abdominal distension scores (33.3 ± 25.5 vs. 54.0 ± 16.4, *p* = 0.014) at 4 weeks compared with placebo. IBS-SSS, clinical response, abdominal pain, and abdominal distension scores were demonstrated (Figures 2, 3).

### 3.3 Quality of life

There was no difference in baseline QOL scores at inclusion in all groups (encapsulated FMT 25.1 ± 5.2 vs. FMT rectal enema 21.0 ± 7.4 vs. placebo 21.6 ± 4.7, *p* = 0.065). Patients in FMT rectal enema group and encapsulated FMT demonstrated a significant improvement of QOL scores at 4 weeks after FMT compared with before FMT by paired *t*-test (FMT rectal enema 30.2 ± 5.0 vs. 21.0 ± 7.4, *p* < 0.001; encapsulated FMT 31.7 ± 4.8 vs. 25.1 ± 5.2, *p* < 0.001), while placebo group had no difference of QOL score (22.4 ± 5.6 vs. 21.6 ± 4.7, *p* = 0.41) (Figure 3). There was a strong negative correlation between IBS-SSS and QOL scores at 4 weeks by Pearson correlation (*r* = −0.64, *p* < 0.001).

### 3.4 Adverse events

There were 6 (40%), 4 (26.7%), and 6 (40%) patients who experienced adverse events in encapsulated FMT, FMT rectal enema, and placebo group, respectively. Minor adverse events such as abdominal pain, bloating, low-grade fever, nausea, and diarrhea, were reported and were not different between all groups (Table 2). Fever and nausea occurred within 24 h after FMT or placebo. All adverse events were self-limiting within 2 days after intervention. No serious adverse event was observed throughout this study.

**Table 2:** Adverse events after receiving FMT or placebo.

**Overall adverse event**	**Encapsulated FMT**	**FMT rectal enema**	**Placebo**	***P*-value**
Abdominal pain	0 (0%)	2 (13.3%)	3 (20.0%)	0.207
Bloating	2 (13.3%)	2 (13.3%)	3 (20.0%)	0.844
Fever	0 (0%)	2 (13.3%)	0 (0%)	0.123
Nausea	1 (6.7%)	2 (13.3%)	1 (6.7%)	0.760
Diarrhea	3 (20.0%)	2 (13.3%)	1 (6.7%)	0.562
**Adverse event within 24 h**	**Encapsulated FMT**	**FMT rectal enema**	**Placebo**	***P*** **-value**
Abdominal pain	0 (0%)	1 (6.7%)	2 (13.3%)	0.343
Bloating	1 (6.7%)	1 (6.7%)	2 (13.3%)	0.760
Fever	0 (0%)	2 (13.3%)	0 (0%)	0.123
Nausea	1 (6.7%)	2 (13.3%)	1 (6.7%)	0.760
Diarrhea	2 (13.3%)	1 (6.7%)	1 (6.7%)	0.760

### 3.5 Microbiome analysis

**Figure 4:**
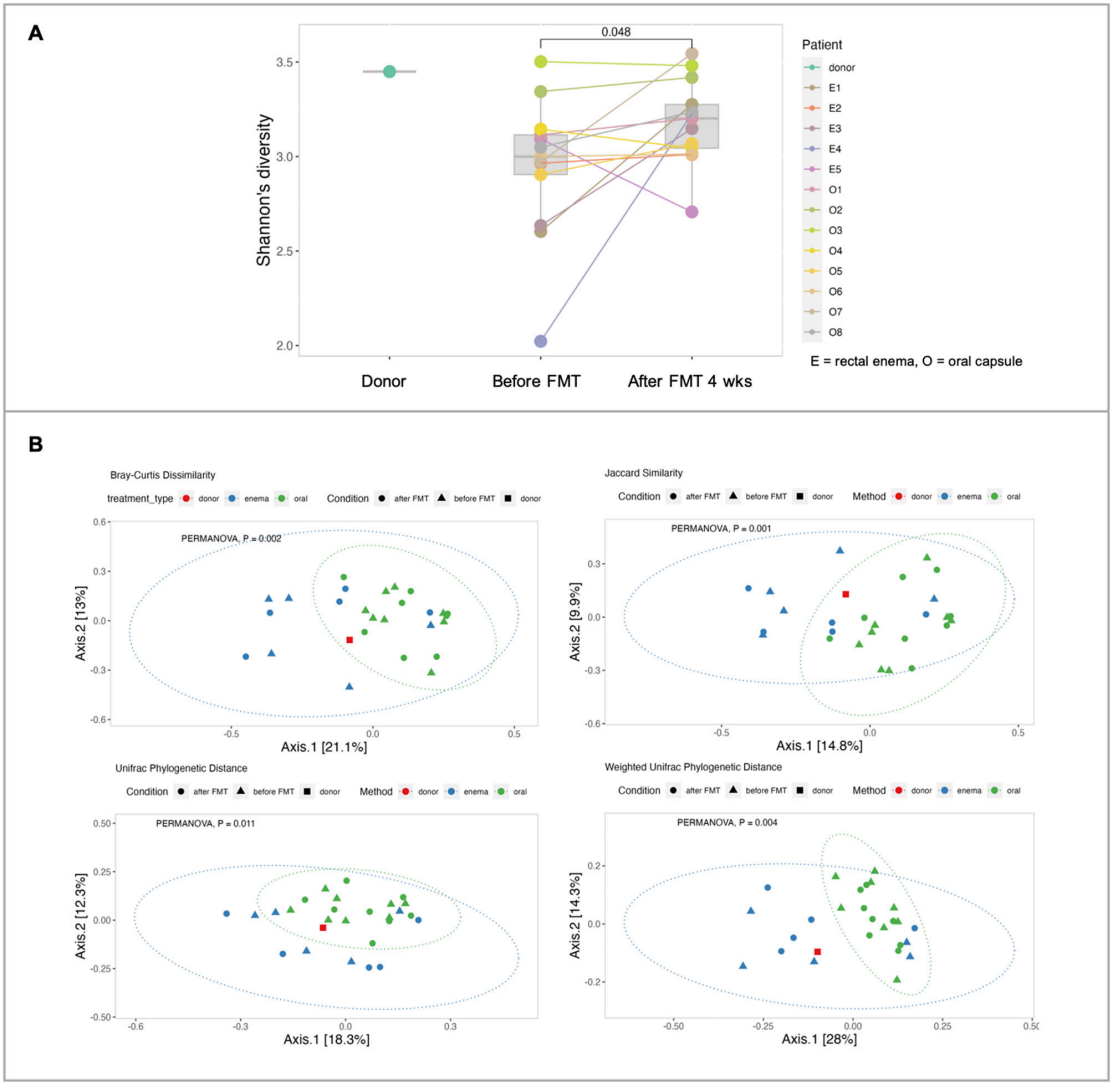
Microbial diversity. **(A)** α-diversities of donors, FMT patients at inclusion (before FMT) and 4 weeks after FMT. **(B)** β-diversities between donor, encapsulated FMT, and FMT rectal enema.

Fecal sample collection was obtained in 13 patients in the FMT group [FMT rectal enema group (*n* = 5) and encapsulated FMT group (*n* = 8)]. Alpha diversity at baseline of patients with IBS was lower than the donor. Analysis of α-diversity demonstrated that Shannon's diversity significantly increased at 4 weeks after FMT compared with before FMT (*p* = 0.048) (Figure 4). Analysis of β-diversity was performed and demonstrated as principal component analysis (PCA) plot based on Bray-Curtis dissimilar matrix, Jaccard similarity, and weighted/unweighted Unifrac phylogenetic distance at species level. There was no difference in β-diversity between before FMT and 4 weeks after FMT (*p* > 0.05) by all methods. However, β-diversity was significantly different between encapsulated FMT and FMT via rectal enema group by Bray-Curtis (*p* = 0.002), Jaccard (*p* = 0.001), weighted Unifrac (*p* = 0.004), and unweighted Unifrac (*p* = 0.011) (Figure 4).

**Figure 5:**
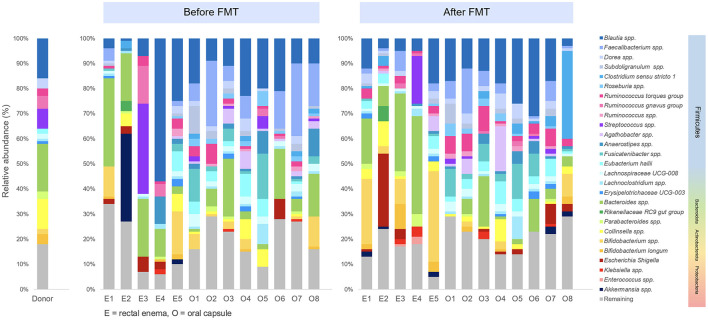
Taxonomic profiles of donor and patients in pre-FMT and post-FMT period.

Differential abundance analysis was performed to determine difference of microbial taxa abundance between before FMT and 4 weeks after FMT. At phylum level, Firmicutes significantly increased (*p* = 0.039), while Bacteriodota remarkably decreased (*p* = 0.0078) at 4 weeks after encapsulated FMT. At genus level, *Dorea* significantly increased after FMT (*p* = 0.013). In encapsulated FMT group, *Ruminococcus torques* increased (*p* = 0.039), whereas *Bacteroides* reduced after FMT (*p* = 0.0078). At species level, *Dorea* spp. (*p* = 0.013), *Bifidobacterium longum* (*p* = 0.044), and *Ruminococcus torques* (*p* = 0.033) significantly increased, while *Faecalibacterium prausnitzii* (*p* = 0.033) significantly decreased after FMT. Taxonomic profiles of donor and patients in pre-FMT and post-FMT period was demonstrated (Figure 5).

## 4 Discussion

In this single-center, double-blind, randomized, placebo-controlled study (RCT), we evaluated the efficacy of encapsulated FMT and FMT via rectal enema to find effective but less invasive routes of administration for patients with IBS. This study demonstrated an improvement in IBS symptoms and quality of life in patients receiving encapsulated FMT and FMT via rectal enema without serious adverse event. Moreover, IBS patients after receiving FMT had significantly higher α-diversity than baseline before FMT.

Our study reported 86.7 and 73.3% overall response rates in encapsulated FMT and FMT via rectal enema group, respectively, with significantly decreased IBS-SSS after treatment. Previous clinical trials of FMT in IBS patients used different delivery routes for FMT including oral capsules (13, 14, 21), upper gastrointestinal routes (e.g., duodenal, nasojejunal) (10, 11, 17), and colonoscopy (12, 22–24). This study is the first RCT comparing the efficacy of FMT via two different routes of administration which are rectal enema and oral capsules. We found that both encapsulated FMT and FMT via rectal enema resulted in better clinical response than placebo. Improved clinical outcome in this study differed from previous trials using FMT capsules which might be due to specific IBS subtypes in other studies (14, 21), different FMT protocol including amount of ingested capsules (13, 14) and antibiotic pretreatment (21). This study used fewer FMT capsules than other trials. However, our capsules were specifically from one donor unlike others using multiple donors (13, 14, 21). Therefore, inter-donor variation of gut microbiota might affect the efficacy of FMT. The result of this study was similar to the study by El-Salhy et al which highlighted an excellent outcome of FMT in IBS patients by using FMT from a single superdonor administered to distal duodenum via gastroscope (10). This suggested that FMT using specimen from a superdonor with satisfactory microbiota profile might induce a donor-dependent clinical response in IBS and inflammatory bowel disease in contrast to *C. difficile* infection which was not affected by specific donors (25, 26). Apart from encapsulated FMT, this study also focused on FMT via rectal enema as a minimally invasive technique. We demonstrated that FMT via rectal enema could provide superior clinical response to placebo at 12 weeks (73.3 vs. 33.3%, *p* = 0.028) which was comparable to previous trials using FMT via colonoscopy (12, 24). Although a response rate of rectal enema was numerically lower than encapsulated FMT (73.3 vs. 86.7%, *p* = 0.651), there was no statistical difference. A slightly lower response rate in rectal enema group might be affected by the time that patient could hold infusate and less colonic distribution to proximal colon (27), whereas patients receiving FMT capsules had an entire amount of FMT distributing in their GI tract. IBS-SSS subscores including abdominal pain and abdominal distension improved after FMT by both oral capsules and rectal enema. Reduction in these symptoms contributed to improved quality of life which was similar to previous studies (10, 11).

Microbiota analysis demonstrated a significant increase in Shannon's diversity index following FMT. Our stool donor had higher microbial diversity than IBS patients at baseline. Therefore, this high-diversity fecal transplant might contribute to successful microbiota engraftment in FMT recipients since key factors predicting engraftment are strain abundance and phylogeny (28). Increased microbial diversity is related to short chain fatty acid production resulting in regulation of immune and inflammatory responses, intestinal barrier integrity, gut motility and maintaining homeostasis in the microbiota-gut-brain axis (29). These mechanisms might explain symptom improvement in IBS in this study. Moreover, this study reported a significant increase in number of specific species after FMT. IBS patients in this study had low relative abundance of *Bifidobacterium* (0–6%) in the pre-FMT period, while our stool donor had higher level of these bacteria (7%). Consequently, a significant increase in *Bifidobacterium longum* after FMT might result in successful outcomes as stated in prior studies that a donor with high abundance of *Bifidobacterium* is a predictor of favorable FMT outcome in IBS patients (15, 30). The previous RCT reported that probiotic *Bifidobacterium longum* NCC3001 could decrease depression scores and increase quality of life in IBS patients by decreasing 4-cresol sulfate and reducing activation of the amygdala and the fronto-limbic complex which involve in mood regulation pathway (31). *Bifidobacterium* might also improve IBS symptom by normalization of the ratio of an anti-inflammatory to a proinflammatory cytokine (32). Our study also demonstrated that *Ruminococcus torques* and *Dorea* spp. increased after FMT. *Ruminococcus torques* is a mucin-degrading bacterium which can cause impaired protective barrier against microbes. Some studies revealed the positive correlation of *Ruminococcus* and *Dorea* with IBS (33, 34), whereas others reported a beneficial effect of enriched *Ruminococcus* in a particular donor (35) and post-FMT patients (36) associated with remission of ulcerative colitis. More than half of patients with increased *Ruminococcus torques* (55.6%) and *Dorea* (66.7%) after FMT were responders in this study. Further research is required to determine the association of these bacteria with IBS symptoms after FMT. Patients with IBS had decreased *Faecalibacterium prausnitzii* (37), a butyrate producer providing benefits to gut health. Our stool donor had a low level of *Faecalibacterium prausnitzii* which could explain a significant decrease of these bacteria in IBS patients after FMT.

Adverse events following FMT were mild and self-limiting without any serious adverse event in this study. Minor adverse events from encapsulated FMT (40%) including abdominal pain, bloating, low-grade fever, nausea, and diarrhea were comparable to those from FMT capsules in previous studies (16.1–84.6%) (13, 14, 21). The number of adverse events from FMT via rectal enema (26.7%) were slightly lower than using FMT via colonoscopy (30.4–50%) (22, 23). Colonoscopy is more invasive than rectal enema and requires bowel preparation which might cause discomfort in IBS patients. Long-term clinical response of both encapsulated FMT and FMT via rectal enema should be followed in future trial.

Our study has several strengths. This double-blind, randomized, placebo-controlled study demonstrated comparative efficacy of different routes of FMT administration in all subtypes of IBS patients. Moreover, this trial suggested less invasive routes of FMT which might be performed to reduce procedural risk from endoscopy. However, the study also had some limitations. Firstly, there were a limited number of patients with different IBS subtypes in each randomized group. Therefore, we could not compare clinical response between each subtype. Future trials with larger sample size are required. Secondly, there were also low number of stool samples for microbiome analysis which could reduce statistical power. Further research is needed to increase sample size to provide more accurate interpretation of microbiome analysis. Thirdly, as IBS is a chronic illness with a fluctuating nature, long-term efficacy as well as delayed adverse effects at 6 or 12 months after FMT require further elucidation. Lastly, although using a single donor has an advantage in reducing interdonor variability, this can result in practical constraints due to insufficient samples in a stool bank causing limitation in general applicability.

In conclusion, this study demonstrated that encapsulated FMT and FMT via rectal enema could provide high efficacy of overall clinical response, IBS-SSS and quality of life in IBS patients without serious adverse event. Increased microbial diversity after FMT might be correlated with favorable outcomes in IBS patients. FMT is a microbiota-based medicine with true clinical potential and should be developed for personalized approach with safer therapeutic outcomes for IBS treatment in the near future.

## Data Availability

The original contributions presented in the study are included in the article/Supplementary material, further inquiries can be directed to the corresponding author.
