# Supplementary material for: Efficacy of encapsulated fecal microbiota transplantation and FMT via rectal enema for irritable bowel syndrome: a double-blind, randomized, placebo-controlled trial (CAP-ENEMA FMT Trial)
Source: Front Med (Lausanne). 2025 Sep 23;12:1648944. doi: 10.3389/fmed.2025.1648944 (PMC12500633; doi:10.3389/fmed.2025.1648944)
Supplement: Supplementary file 1 [file Table_1.docx]

**Supplementary Table 1.** Laboratory testing for stool donor screening

| **Blood tests** | **Stool tests** |
| --- | --- |
| - Complete blood count - Blood urea nitrogen - Creatinine - Electrolyte - Liver function test - Blood sugar - C-reactive protein - Anti-HIV 4th generation test - Anti-HAV IgM - HBsAg, Anti-HBc IgM - Anti-HCV - *Strongyloides* IgG antibody - Anti-*Helicobacter pylori* IgG - VDRL | - Stool exam (3 days) - Stool AFB (3 days) - Stool modified AFB (3 days) - Stool concentration for parasites (3 days) - Stool culture for   - Enteric pathogen (*Salmonella, Shigella, Campylobacter, Vibrio, E. coli* Shiga toxin)   - Vancomycin-Resistant Enterococci (VRE)   - Extended-spectrum beta-lactamase-producing gram-negative rods (ESBL)   - Carbapenemase-producing gram-negative rods (CRE) - Stool for *C. difficile* toxin A & B and glutamate dehydrogenase immunoassay - Stool PCR for *C. difficile* - Stool PCR for Norovirus - Stool PCR for SARS-CoV-2 - Stool EIA for Adenovirus/Rotavirus - Stool EIA for *Cryptosporidium* - Stool EIA for *Helicobacter pylori* antigen |

AFB = Acid-fast bacillus, EIA = Enzyme Immunoassay, PCR = Polymerase chain reaction, VDRL = Venereal Disease Research Laboratory test
